# Pathological mechanisms and novel drug targets in fibrotic interstitial lung disease

**DOI:** 10.1186/s41232-024-00345-2

**Published:** 2024-07-19

**Authors:** Yasuhiko Nishioka, Jun Araya, Yoshiya Tanaka, Atsushi Kumanogoh

**Affiliations:** 1 Tokushima University, Tokushima, Japan; 2 The Jikei University School of Medicine, Tokyo, Japan; 3 University of Occupational and Environmental Health, Kitakyushu, Japan; 4 Department of Respiratory Medicine and Clinical Immunology, Graduate School of Medicine, Osaka University, 2-2 Yamada-oka, Suita, Osaka 565-0871 Japan

**Keywords:** Fibrosis, Interstitial lung disease, Progressive, Pathological mechanisms, Drug targets, PDE4, cAMP

## Abstract

**Background:**

Interstitial lung diseases (ILDs) are a diverse group of conditions characterized by inflammation and fibrosis in the lung. In some patients with ILD, a progressive fibrotic phenotype develops, which is associated with an irreversible decline in lung function and a poor prognosis.

**Main body:**

The pathological mechanisms that underlie this process culminate in fibroblast activation, proliferation, and differentiation into myofibroblasts, which deposit extracellular matrix proteins and result in fibrosis. Upstream of fibroblast activation, epithelial cell injury and immune activation are known initiators of fibrosis progression, with multiple diverse cell types involved. Recent years have seen an increase in our understanding of the complex and interrelated processes that drive fibrosis progression in ILD, in part due to the advent of single-cell RNA sequencing technology and integrative multiomics analyses. Novel pathological mechanisms have been identified, which represent new targets for drugs currently in clinical development. These include phosphodiesterase 4 inhibitors and other molecules that act on intracellular cyclic adenosine monophosphate signaling, as well as inhibitors of the autotaxin-lysophosphatidic acid axis and $\alpha_v$ integrins. Here, we review current knowledge and recent developments regarding the pathological mechanisms that underlie progressive fibrotic ILD, including potential therapeutic targets.

**Conclusion:**

Knowledge of the pathological mechanisms that drive progressive fibrosis in patients with ILD has expanded, with the role of alveolar endothelial cells, the immune system, and fibroblasts better elucidated. Drugs that target novel mechanisms hold promise for expanding the future therapeutic armamentarium for progressive fibrotic ILD.

## Background

Interstitial lung disease (ILD) is an umbrella term for a pathologically broad group of conditions characterized by inflammation and fibrosis in the functional tissue of the lung [1, 2]. Some patients with ILDs exhibit a progressive fibrotic phenotype associated with worsening symptoms, an irreversible decline in lung function, and a poor prognosis [1, 2]. Idiopathic pulmonary fibrosis (IPF), the most severe fibrotic ILD, is often considered synonymous with the phenotype of fibrosing ILD [1, 3]. However, irreversible progressive fibrosis also presents in patients with other types of ILDs [2, 3].

Two antifibrotic drugs, nintedanib and pirfenidone, are approved for use in IPF [4], with growing evidence that they may also delay the progression of fibrosis in other progressive fibrotic ILDs, recently referred to as progressive pulmonary fibrosis [2]. These agents act predominantly by inhibiting fibroblast migration, activation, and differentiation [1, 5, 6]. Although fibroblasts play a central role in the pathology of IPF and other fibrotic ILDs, various other tissues and diverse cell populations are involved in disease mechanisms that occur upstream of fibroblasts [7]. The understanding of these pathological processes has expanded in recent years, in part due to the advent of single-cell RNA sequencing technology, which facilitates detailed analysis of the transcriptome of specific cells within a tissue [8, 9], as well as integrative multiomics analyses that allow high-throughput assessment of genomic, transcriptomic, and/or proteomic changes (as successfully applied in rheumatoid arthritis [RA], for example) [8, 10, 11]. This enhanced knowledge is facilitating the development of therapies for ILDs with novel mechanistic targets, such as phosphodiesterase (PDE) 4 and other modulators of intracellular cyclic adenosine monophosphate (cAMP) signaling [12, 13], as well as inhibitors of the autotaxin (ATX)-lysophosphatidic acid (LPA) axis [14] and α_v_ integrins [15].

The objective of this review is to reflect on the current understanding of the pathogenesis of progressive fibrotic ILD, focusing on the role of diverse epithelial and immune cell populations, as well as fibroblasts. In addition, the mechanisms and targets of investigative therapies with novel modes of action that hold promise for the treatment of progressive fibrotic ILD are described.

### Classification and diagnosis of primary diseases in fibrotic ILD

**Fig. 1:**
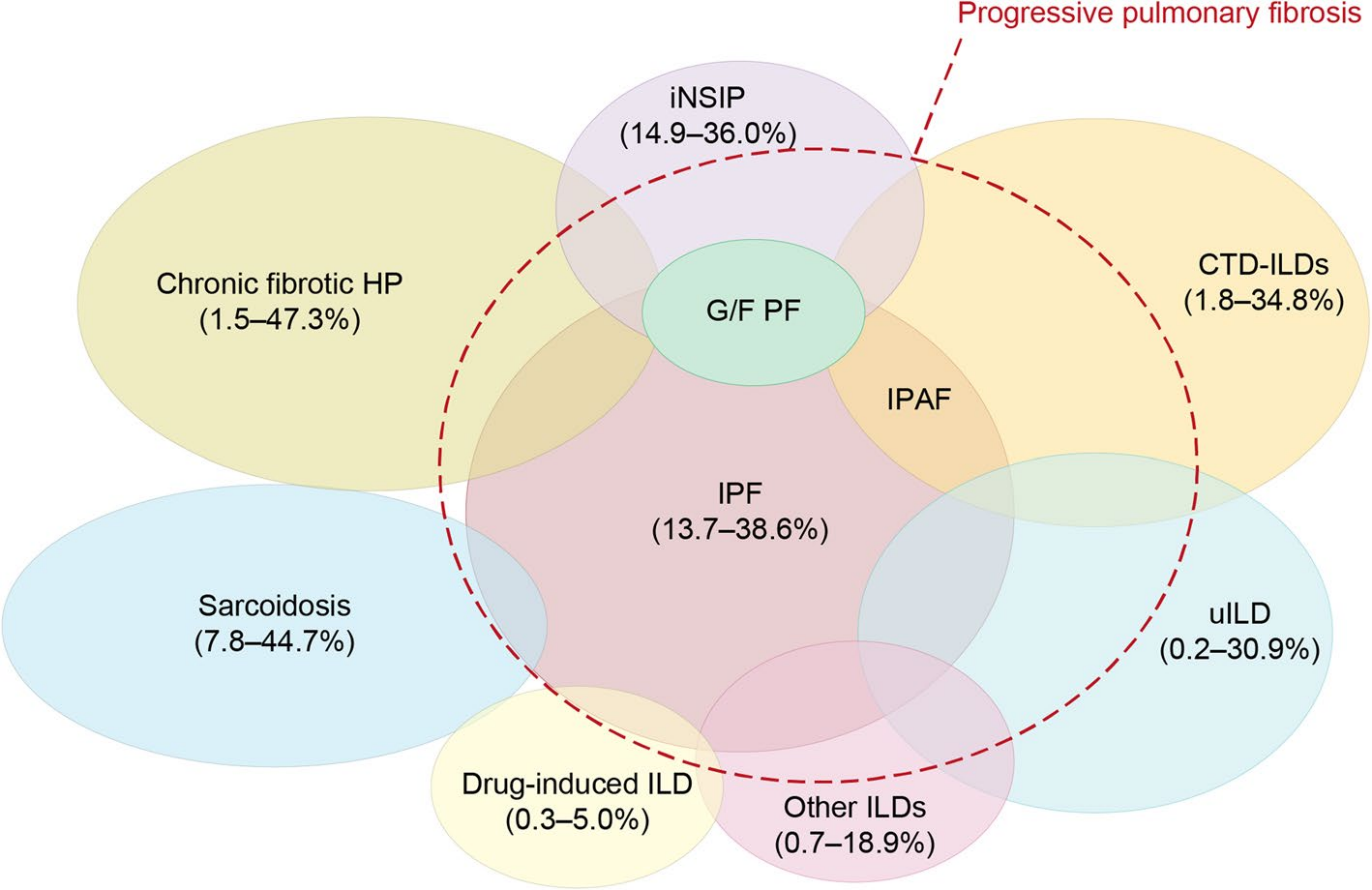
The prevalence of ILDs may be associated with progressive pulmonary fibrosis (despite management). Reproduced from Rajan et al. (2023) [2] under the terms of the Creative Commons Attribution Non-Commercial Licence 4.0. Abbreviations: CTD-ILD connective tissue disease interstitial lung disease, G/F PF genetic and/or other familial pulmonary fibrosis, HP hypersensitivity pneumonitis, ILD interstitial lung disease, iNSIP idiopathic nonspecific interstitial pneumonia, IPAF interstitial pneumonia with autoimmune features, IPF idiopathic pulmonary fibrosis, uILD unclassifiable ILD

There are multiple diverse types of fibrotic ILD (Fig. 1), including those that have a known trigger, such as underlying connective tissue disease (CTD) or environmental exposure to various substances, and those that are idiopathic in nature, comprising the idiopathic interstitial pneumonias (IIP) such as IPF [2]. The most common primary progressive fibrotic ILD across Europe and the USA is IPF (estimated prevalence 1.3–16.7 cases/100,000 people) followed by sarcoidosis (0.2–4.5 cases/100,000), with the overall prevalence of ILDs with a progressive fibrosing phenotype estimated at 2.2–28.0 cases/100,000 people [16]. In Japan, the estimated prevalence of IPF from 2003 to 2007 was 10.0/100,000 people based on a medical claims database analysis in Hokkaido [17]. A more recent report indicates that estimates are increasing in Japan, with an estimated prevalence of 27.0/100,000 from 2017 to 2018 [18]. However, it appears that sarcoidosis may be less common in patients from Japan and Korea than in those from Western countries, with the most common progressive fibrotic ILDs after IPF being CTD-associated ILDs and unclassifiable IIP [19, 20].

**Fig. 2:**
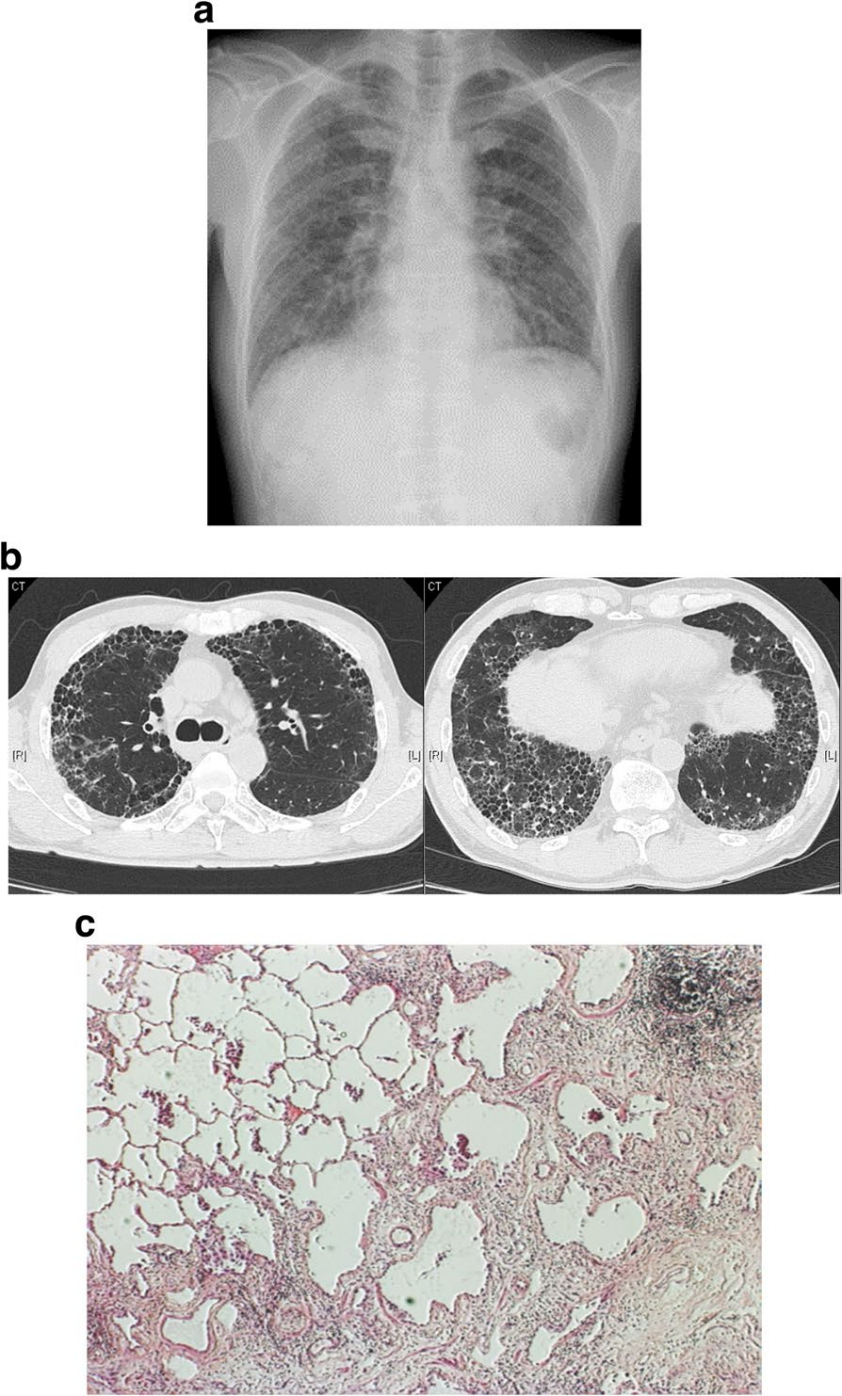
Representative (**a** and **b**) lung images in IPF and (**c**) histology illustrating features of UIP. Shown are representative **a** chest X-ray and **b** HRCT scans of the lungs of a patient with IPF, and **c** histology illustrating features of UIP. Images **a** and **b** were reproduced from Imakura et al. (2020) with permission [24]. Image **c** was provided by Y Nishioka. Abbreviations: HRCT high-resolution computed tomography, IPF idiopathic pulmonary fibrosis, UIP usual interstitial pneumonia

Identification of fibrotic ILD centers on radiological assessment by high-resolution computed tomography (HRCT), in which peripheral, lower-lobe predominant traction bronchiectasis is suggestive of IPF and chest X-ray [21, 22]. The histopathological pattern is that of usual interstitial pneumonia (UIP), characterized by the presence of honeycombing [23, 24] (Fig. 2). During progression of IPF, the proportion of the lung exhibiting the UIP pattern expands in both the transverse and coronal planes, and honeycomb cysts often increase in size and number [21]. HRCT and chest X-ray are also used to identify progressive fibrosis in other types of ILDs [21, 22]. Although there is variation in the changes that occur, the following features are indicative of progressive fibrosis: an increase in traction bronchiectasis/bronchiectasis, coarseness of reticular abnormality, and lobar volume loss, or the development of new ground-glass opacity with traction bronchiectasis, new fine reticulation, and new/increased honeycombing [21].

It is important to consider that ILDs are characterized to a varying extent by inflammation and fibrosis, and thus patients may present with a mainly inflammatory phenotype at one end of the spectrum (potentially responding well to immunosuppression and anti-inflammatory treatment), a mostly fibrotic disease at the other extreme (with a poor prognosis and limited therapeutic options), or varying degrees of both inflammation and fibrosis [25]. IPF sits at the fibrotic end of this continuum, whereas fibrosis in non-IPF ILDs is often triggered by, or otherwise linked to, inflammation [1].

### Pathological mechanisms of fibrotic ILD

Although fibrosis is a normal physiological response that occurs as part of wound healing and host defense against pathogens [26], this process can malfunction, causing exaggerated pro-inflammatory and profibrotic responses [7]. This leads to fibroblast activation and differentiation into myofibroblasts, extracellular matrix (ECM) deposition, and remodeling of the functional lung tissue, resulting in tissue stiffness and compromised gaseous exchange [7, 9]. Potential therapeutic targets at any stage of fibrotic lung disease include not only fibroblasts, but also the pathological processes that occur upstream of these mediators [6]; thus, it is important to elucidate mechanisms that lead to fibroblast activation in ILD that are, as yet, not fully understood.

**Fig. 3:**
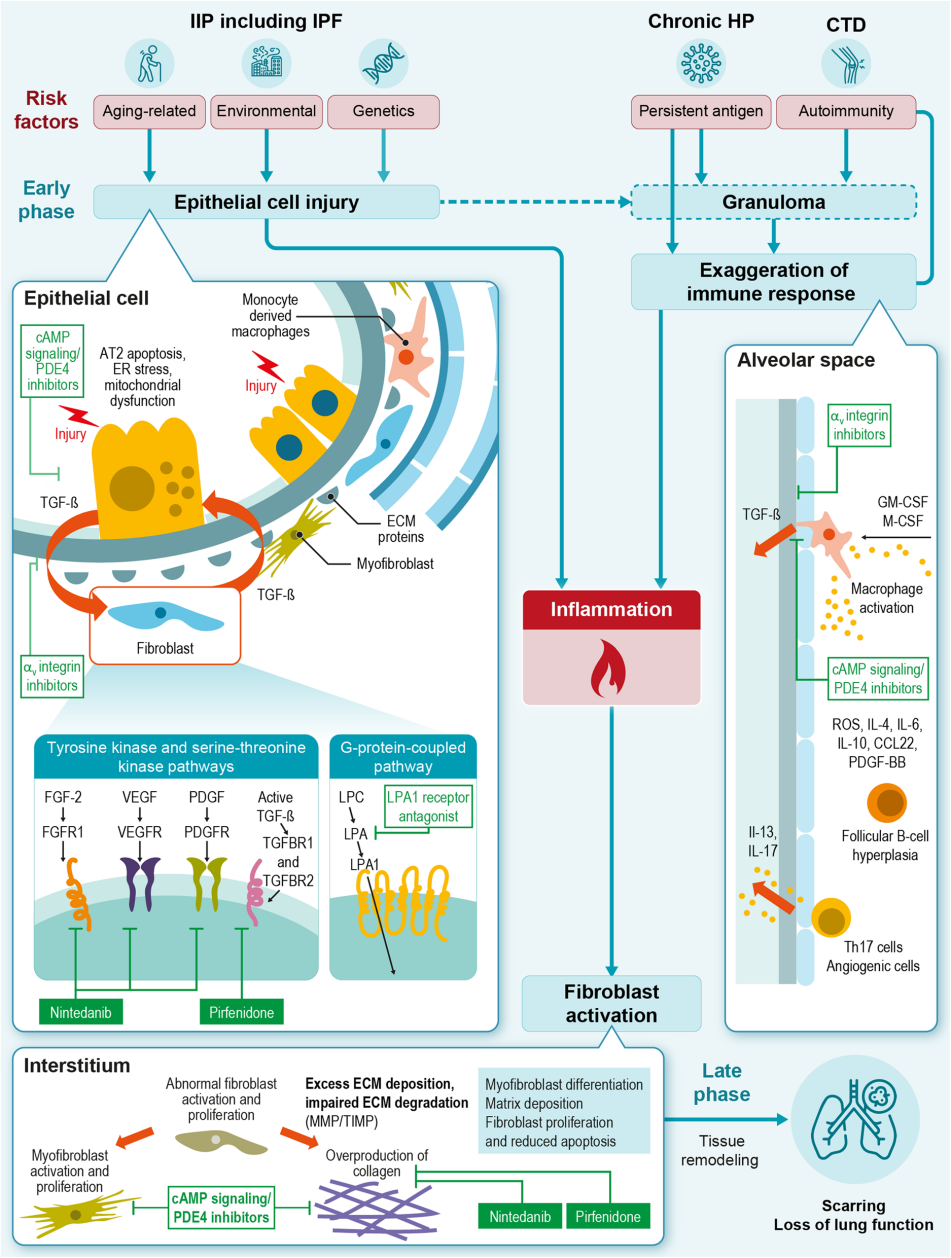
Mechanistic overview of the pathogenesis of primary pulmonary fibrosis with therapeutics and their targets. The figure was created based on information in Wijsenbeek et al. (2020), Justet et al. (2022), Kolb et al. (2023), Decaris et al. (2021), Chiang et al. (2023), Zhao et al. (2022), Ruwanpura et al. (2020), Zulfikar et al. (2020), and Lederer et al. (2018) [7, 9, 12, 15, 28–32]. Filled green boxes with white text (approved drugs) and transparent boxes with green text (candidate drugs). Abbreviations: cAMP cyclic adenosine monophosphate, CCL22 C-C motif chemokine 22, CTD connective tissue disease, ECM extracellular matrix, ER endoplasmic reticulum, FGF fibroblast growth factor, FGFR fibroblast growth factor receptor, GM-CSF granulocyte-macrophage colony-stimulating factor, HP hypersensitivity pneumonitis, IIP idiopathic interstitial pneumonia, IL interleukin, IPF idiopathic pulmonary fibrosis, LPA lysophosphatidic acid, LPC lysophosphatidylcholine, M-CSF macrophage colony-stimulating factor, MMP matrix metalloproteinase, PDE phosphodiesterase, PDGF platelet-derived growth factor, PDGF-BB platelet-derived growth factor BB, PDGFR platelet-derived growth factor receptor, ROS reactive oxygen species, TGF-β transforming growth factor β, TGFBR transforming growth factor beta receptor, TIMP tissue inhibitor of metalloproteinase, VEGF vascular endothelial growth factor, VEGFR vascular endothelial growth factor receptor

The initial triggers of fibrotic ILD can be broadly categorized into two types (Fig. 3, top): epithelial cell injury, as is the case of IIPs such as IPF, and immune activation, which can result from autoimmune disease (as occurs in CTD-associated ILDs) or exposure to a persistent antigen (as occurs in chronic fibrotic hypersensitivity pneumonitis [HP]) [7, 27].

### Initiation of the fibrotic process through epithelial cell injury

Epithelial cell injury is an important initial step in the progression of lung fibrosis, particularly in IPF [7, 27], and has multiple potential triggers, including environmental exposure from smoking, occupational hazards, air pollution, and viruses [7]. These triggers cause alveolar epithelial cell injury, which along with compromised cellular repair mechanisms drive disease progression [27]. Recent advances in single-cell RNA sequencing technology are furthering the understanding of how diverse epithelial cell populations in the human lung contribute to this process [33]. These studies have highlighted marked shifts in epithelial cell populations in IPF lungs following repeated injury, including an increased proportion of airway epithelial cells and a decrease in alveolar epithelial cells [34], indicating “proximalization” of the distal lung [9]. A population of aberrant basaloid cells has been discovered, which express basal epithelial, mesenchymal, and senescence markers, as well as developmental transcription factors [34, 35]. These cells may be involved in driving pulmonary fibrosis, as they express a multitude of genes implicated in the pathogenesis of IPF [9].

Overall, the changes in epithelial cellular composition lead to abnormal secretion of profibrotic mediators [9] (Fig. 3, top left). These include transforming growth factor (TGF)-β1, platelet-derived growth factor (PDGF), fibroblast growth factors (FGFs), connective tissue growth factor, tumor necrosis factor (TNF), endothelin-1, CXC chemokine ligand 12, and osteopontin [27, 36]. The secretion of these factors from alveolar epithelial cells drives the process of fibroblast migration, proliferation, activation, and differentiation into myofibroblasts that results in lung fibrosis [27].

### Genetic mechanisms and the role of cellular senescence and autophagy

A multitude of genetic and cellular changes contribute to progressive fibrosis in ILD. Genomic studies have unveiled some genetic mechanisms that may be involved [7, 37]. A common gain-of-function variant in the promoter of *MUC5B*, which encodes mucin 5B, a glycoprotein component of airway mucus with a role in airway clearance and defense against bacterial infection, has been identified as a risk factor for different types of fibrotic ILD, including IPF, RA-ILD, and chronic HP [7, 38]. Notably, the frequency of this variant is heterogeneous, and is more common in European versus Japanese people—this illustrates the importance of conducting genomic analysis across multiple populations [38]. A recent genome-wide association study of Japanese patients with RA-ILD identified *RPA3-UMAD1* at 7p21 as a novel risk locus; this gene encodes replication protein A3, which is involved in cellular response to DNA damage and telomere elongation [39]. Protein-altering variants in other telomere-related genes have also been identified in patients with a range of ILDs, including IPF, chronic HP, and RA-ILD, supporting a pathogenic role of telomere dysfunction in progressive fibrosis [6, 37, 40].

Related to these findings, cellular senescence, a state of non-reversible cell cycle arrest that can be induced by telomere attrition, has been identified as a significant contributor to the pathophysiology of UIP, irrespective of underlying etiology [41, 42]. Senescence is typically induced by persistent DNA damage, telomere attrition, and stress signaling, including oxidative stress triggered by excessive generation of reactive oxygen species [41]. Epithelial cells in remodeled areas of lung tissue from patients with UIP express p16 and p21, markers of cell senescence [42], and these senescent cells secrete pro-inflammatory molecules, including interleukin (IL)-1a, IL-1b, IL-6, and IL-10, as well as TGF-β [42]. Certain markers of autophagy, a cellular process for degradation of cellular debris, are also upregulated in epithelia and myofibroblasts in UIP lung tissue, likely in response to local hypoxia [42]. It has been shown that autophagy is necessary for TGF-β-induced fibrosis in IPF [43]. However, the role of autophagy in the pathogenesis of pulmonary fibrosis remains to be fully explored [44].

### Potential biomarkers for fibrotic ILDs

A greater understanding of the mechanisms that drive progressive fibrosis in ILD has led to the identification of potential biomarkers. For example, Krebs von den Lungen-6 (KL-6) and alveolar surfactant protein subtypes A and D (SP-A and SP-D) have been shown to discriminate various types of ILD from non-ILD controls and to predict disease prognosis [45]. The presence of KL-6 in serum indicates the pathological release of this protein into the blood from pneumocytes following injury to bronchiolar epithelial cells and the basement membrane [46]. SP-A and SP-D are involved in innate immunity and in the opsonization and lysis of inhaled pathogens [46, 47]. Findings from a meta-analysis have indicated that KL-6 may have greater diagnostic accuracy than SP-D for differentiating ILD from non-ILD among patients with CTD [41]. Although the performance of individual biomarkers is currently not generally sufficient for clinical use [45], the increasing availability of high-throughput proteomic analyses is anticipated to identify novel diagnostic and prognostic biomarkers in the future [45]. For example, a recent multiomic analysis has facilitated the identification of a molecular endotype of patients with IPF who are at greater risk of disease progression [10].

### Initiation of the fibrotic process through immune activation

Immune activation can be the primary trigger for disease pathogenesis in many types of fibrotic ILDs [28, 48]. CTDs cause chronic inflammation and dysregulated immunity, leading to the activation of diverse immune cell populations and the production of cytokines that modulate fibroblast activation [28, 49] (Fig. 3, top right). Various types of CTDs are associated with fibrotic ILDs, such as RA, systemic sclerosis (SSc), idiopathic inflammatory myopathy, and rarely, Sjögren’s syndrome [6]. Activated macrophages have been reported to accumulate in SSc-ILD lungs, and pro-inflammatory Th2 cytokines such as IL-4, IL-5, and IL-13 have been found at higher levels than in healthy controls [50, 51]. B cells may also play a role, with extensive B cell infiltration noted in the lung tissue of patients with SSc-ILD [28, 48].

### The role of inflammation

Aberrant immune activation can also lead to fibrosis in the setting of chronic HP, but in this case, it is the exposure to one or more antigens that instigates the inflammatory disease process [7, 52]. Diverse inciting agents have been reported, ranging from protein antigens derived from microorganisms to non-protein chemicals [52]. Both humoral and Th1 cellular immune responses are activated following antigen exposure, leading to lymphocytic and granulomatous inflammation [52] (Fig. 3, bottom).

The extent to which mechanisms of immunopathogenesis differ across distinct types of fibrotic ILD is not well understood. Certain commonalities have been uncovered, such as the presence of monocyte-derived profibrotic alveolar macrophages identified by single-cell RNA sequencing in various types of fibrotic ILDs, including IPF, SSc-ILD, and chronic HP [9, 33]. However, differences also exist, for example, between the B cell and T cell chemokine profiles of IPF and SSc-ILD [6]. More B cells and CD4 T cells are found in the lungs of people with RA-ILD compared with idiopathic UIP, suggesting greater immune dysregulation in the former [6]. In IPF, although the immune system and inflammation are thought to play a role in disease pathogenesis, with recruitment and activation of immune cells known to modulate the established fibrotic response, the exact mechanisms involved require further elucidation [53–56]. Single-cell RNA sequencing is expanding knowledge in this area. Elevated numbers of alveolar macrophages, dendritic cells, and CD4 and CD8 memory T cells have been found in the lungs of patients with IPF compared with healthy controls [56]. Transcriptomic profiling revealed the expression of genes associated with interferon-γ response in these cells, along with the upregulation of genes related to adaptive immunity [56]. Further studies are needed to fully elucidate these mechanisms and the mechanistic differences and similarities between fibrotic ILDs.

Although our article focuses on the pathogenic mechanisms that drive the chronic progression of fibrotic ILD, it is important to note that the clinical course of ILD can also exhibit acute deterioration. This can take the form of a de novo rapidly progressive ILD or an acute exacerbation of existing ILD [57–59], and is characterized by severe deterioration in respiratory function over a short time period that can be life-threatening [1, 60]. Various triggers of acute exacerbation of ILD have been identified, but it can also be idiopathic [60]. The mechanisms that underlie rapidly progressive forms of ILD, such as acute interstitial pneumonia, are not fully established and beyond the scope of this paper [59]. Misconceptions exist around the clinical and pathological differences between fibrotic ILD and rapidly progressive ILD or acute exacerbations of ILD, which have important implications for treatment choice, as discussed later in this review. Greater standardization in the terminology used for the different forms of ILD may help to address this.

### Pathogenic mechanisms in fibrotic ILD downstream of fibroblast activation

Above, we have considered how alveolar epithelial cell injury and immune dysregulation can initiate the pathogenesis of fibrotic ILD by creating a profibrotic environment. The result of these processes is fibroblast activation and differentiation into myofibroblasts [7], which produce ECM proteins that progressively restructure and stiffen the lung architecture [6] (Fig. 3, bottom). As well as excess ECM deposition, impaired ECM degradation contributes to its accumulation in fibrotic lungs [29]. The primary molecules involved in regulating ECM degradation are the matrix metalloproteinases (MMPs) and the tissue inhibitors of metalloproteinases (TIMPs) [29]. ECM components, such as collagen, fibronectin, laminin, and gelatin, are cleaved into small peptides by MMPs, and the balance of MMPs and TIMPs determines the degree of protein hydrolysis [29]. Among the multiple members of the MMP family, diverse effects on pulmonary fibrosis have been uncovered [61]. Profibrotic isotypes include MMP-3, MMP-8, MMP-11, and MMP-28, whereas MMP-10 and MMP-19 are antifibrotic [61]. Some MMPs have been identified as both profibrotic and antifibrotic, whereas the effect of other MMPs is unknown [61]. MMPs have also been recognized as potential biomarkers for the diagnosis of IPF [62].

### Mechanisms of action of currently approved antifibrotic therapies

Two antifibrotic drugs, pirfenidone and nintedanib, are approved for the treatment of IPF, and nintedanib is also approved for the treatment of other chronic fibrosing ILDs with a progressive phenotype and SSc-ILD [63–66]. As antifibrotic therapies are not suitable for the management of rapidly progressive ILD or acute exacerbations of ILD [21, 67], misconceptions around the different types of ILD can lead to inappropriate prescribing.

Nintedanib is a potent inhibitor of the receptor tyrosine kinases PDGF receptor-α, PDGF receptor-β, FGF receptor-1, FGF receptor-2, FGF receptor-3, FGF receptor-4, vascular endothelial growth factor receptor-1, vascular endothelial growth factor receptor-2, and vascular endothelial growth factor receptor-3, and of the Src family of non-receptor tyrosine kinases [5]. Nintedanib inhibits the activity of these kinases by binding to the intracellular adenosine triphosphate (ATP)-binding pocket [5]. In vitro, nintedanib inhibits PDGF receptor-α-mediated fibroblast proliferation, PDGF- and FGF-stimulated fibroblast motility, and TGF-β-induced fibroblast to myofibroblast transformation [5]. Nintedanib also decreases TGF-β-stimulated collagen secretion and deposition by lung fibroblasts in vitro, potentially by reducing TIMP-2 and inducing MMP-2 secretion [5]. In rats with bleomycin-induced fibrosis, nintedanib inhibits mRNA expression of TGF-β1 and procollagen 1 and reduces collagen deposition in the lung [5]. Nintedanib is also thought to have anti-inflammatory effects [5], as well as inhibitory effects on immune cells [68, 69]. In summary, the antifibrotic effects of nintedanib are due to its ability to impede fibroblast proliferation, migration, and differentiation, as well as to reduce the secretion of ECM proteins [5].

Although the mechanism of action of pirfenidone is not as fully understood, similarly to nintedanib, it also inhibits fibroblast migration, activation, and differentiation, and has anti-inflammatory properties [1, 6]. Pirfenidone suppresses TGF-β1 mRNA expression and protein levels, along with TGF-β1/SMAD-3-induced fibroblast proliferation and differentiation, and inhibits PDGF-α and PDGF-β synthesis, and basic FGF expression [30]. In vivo, pirfenidone reduces collagen content in the lung and decreases prolyl hydroxylase activity, a marker of collagen synthesis [70]. Pirfenidone can also modulate the expression of the MMP/TIMP system, which is involved in the regulation of ECM degradation [30]. In addition, pirfenidone decreases the expression of pro-inflammatory cytokines, such as TNF-α [30], and markers of oxidative stress in the lungs [70].

Rather than antifibrotic therapy, the management of rapidly progressive ILD centers around the use of glucocorticoid therapy and immunosuppressants [21, 58, 67]. For example, conditional treatment recommendations from the American College of Rheumatology recommend against the use of nintedanib and pirfenidone for rapidly progressive ILD associated with systemic autoimmune diseases, instead recommending that first-line treatment comprises glucocorticoids plus one or two other immunosuppressants, with consideration of early referral for lung transplantation [67]. Antifibrotic therapy is recommended after standard treatments have failed [21, 58, 67].

### Novel therapeutic targets in fibrotic ILD

There are several drug candidates in clinical development for fibrotic ILD that have novel modes of action. One such mechanistic target is enhanced intracellular signaling by the second messenger cAMP [12]. Inhibitors of PDE4B hold promise in this regard. PDE4B is an enzyme that mediates the hydrolysis of cAMP; its inhibition leads to elevated intracellular cAMP levels and activation of protein kinase A, as well as exchange protein directly activated by cAMP (EPAC1) [12]. EPAC1 appears to downregulate inflammatory signaling, in part by enhancing barrier function and limiting permeability of vascular endothelial cells, as well as promoting the release of anti-inflammatory cytokines and decreased release of pro-inflammatory cytokines [12, 71]. PDE4B inhibition can also inhibit lung neutrophil influx in vivo [72]. The signaling mechanisms by which increased cAMP initiates antifibrotic effects are not well understood [12], but it has been shown that PDE4B inhibition reduces the expression of fibrogenic genes [73], blocks TGF-β-induced myofibroblast transformation [72], decreases the expression of ECM proteins [12], and reduces collagen content in the lungs [73]. PDE4 inhibition has also been shown to promote the integrity of the alveolar epithelium [73]. Nerandomilast (BI 1015550) is a PDE4B inhibitor that is under investigation in phase 3 clinical trials for the treatment of IPF [12]. Another PDE4 inhibitor under investigation, AA6216, was shown to attenuate pulmonary fibrosis in mice by inhibiting the release of profibrotic cytokines from macrophages, including TGF-β1—which induces fibroblast differentiation to myofibroblasts—and TNF-α [74]. Another drug being investigated in phase 3 trials for IPF, inhaled treprostinil [75], is a prostacyclin analogue that also has a mode of action that involves enhanced cAMP signaling [76]. Treprostinil binds to the prostaglandin E receptor 2, the prostacyclin receptor, and the prostaglandin D receptor 1, which triggers the activation of adenylate cyclase and the conversion of ATP to cAMP, leading to downstream antifibrotic effects [76].

Inhibition of the ATX-LPA axis is another novel therapeutic target in fibrotic ILD [31]. LPA is a phospholipid growth factor that binds to LPA receptors, eliciting diverse downstream functions [31], whereas ATX is a secreted enzyme that generates the majority of circulating LPA through hydrolysis [31]. LPA signaling is involved in the development of lung fibrosis in multiple ways, including stimulating fibroblast activation, proliferation, and migration, and promoting IL-8 secretion and an inflammatory response [14, 31]. In addition, LPA signaling triggers epithelial cell apoptosis and increased vascular permeability, and may therefore play a role in the disruption of the alveolar-capillary membrane [31]. Admilparant (BMS-986278) is an LPA receptor 1 antagonist [14] that decreased the rate of lung function decline in a phase 2 trial in patients with IPF or progressive fibrotic ILD [77, 78], and is subsequently entering phase 3 development (clinicaltrials.gov, NCT06025578 and NCT06003426). Additionally, two ATX inhibitors, BBT-877 and cudetaxestat (BLD-0409), are currently undergoing phase 2 trials (clinicaltrials.gov, NCT05483907 and NCT05373914).

Another mechanism of therapeutic interest in fibrotic ILD is the inhibition of α_v_ integrins, which are transmembrane proteins implicated in the conversion of TGF-β from its inactive to active form [15]. Dual inhibition of the α_v_β_6_ and α_v_β_1_ integrins with bexotegrast (PLN-74809) reduced collagen gene expression ex vivo in lung tissue from patients with IPF and inhibited collagen deposition in vivo in bleomycin mouse models [15]. A phase 2a clinical trial of bexotegrast, an oral, small molecule, dual-selective inhibitor of α_v_β_6_ and α_v_β_1_, in patients with IPF has shown promising results, and a phase 2b trial is planned [79].

## Conclusions

Knowledge of the pathological mechanisms that drive progressive fibrosis in patients with ILD has expanded, with the role of alveolar endothelial cells, the immune system, and fibroblasts better elucidated due to technological advances in sequencing and multiomic analyses. These insights have uncovered novel pathogenic targets with diverse downstream effects, including modulation of inflammatory response and alveolar epithelial cell integrity, in addition to their impact on fibroblasts. There are now several drugs in clinical development that target these novel mechanisms and hold promise for expanding the future therapeutic armamentarium for progressive fibrotic ILD.

## Data Availability

Data sharing is not applicable to this article as no datasets were generated or analyzed during the current study.

## Abbreviations

ILD: Interstitial lung disease
IPF: Idiopathic pulmonary fibrosis
PDE: Phosphodiesterase
RA: Rheumatoid arthritis
cAMP: Cyclic adenosine monophosphate
ATX: Autotaxin
LPA: Lysophosphatidic acid
CTD: Connective tissue disease
IIP: Idiopathic interstitial pneumonia
HRCT: High-resolution computed tomography
UIP: Usual interstitial pneumonia
ECM: Extracellular matrix
HP: Hypersensitivity pneumonitis
TGF: Transforming growth factor
PDGF: Platelet-derived growth factor
FGF: Fibroblast growth factor
TNF: Tumor necrosis factor
IL: Interleukin
KL-6: Krebs von den Lungen-6
SP-A: Surfactant protein subtype A
SP-D: Surfactant protein subtype D
SSc: Systemic sclerosis
MMP: Matrix metalloproteinase
TIMP: Tissue inhibitor of metalloproteinase
ATP: Adenosine triphosphate
EPAC1: Exchange protein directly activated by cAMP
G/F PF: Genetic and/or other familial pulmonary fibrosis
iNSIP: Idiopathic nonspecific interstitial pneumonia
IPAF: Interstitial pneumonia with autoimmune features
uILD: Unclassifiable ILD
CCL22: C-C motif chemokine 22
ER: Endoplasmic reticulum
GM-CSF: Granulocyte-macrophage colony-stimulating factor
LPC: Lysophosphatidylcholine
M-CSF: Macrophage colony-stimulating factor
ROS: Reactive oxygen species
VEGF: Vascular endothelial growth factor
CTD-ILD: Connective tissue disease interstitial lung disease
